# Supplementary material for: Pyruvate Dehydrogenase Kinase 1 inhibition mediated oxidative phosphorylation enhancement in cartilage promotes osteoarthritis progression
Source: BMC Musculoskelet Disord. 2023 Jul 20;24:597. doi: 10.1186/s12891-023-06585-6 (PMC10357736; doi:10.1186/s12891-023-06585-6)
Supplement: Supplementary file 1 — Additional file 1. [file 12891_2023_6585_MOESM1_ESM.zip › KEGG.Gsea.1653622432464/KEGG_PEROXISOME.html]

Details for gene set KEGG\_PEROXISOME[GSEA]

|  || Dataset | 55457\_collapsed\_to\_symbols.cls.cls #OA\_versus\_normal.cls.cls #OA\_versus\_normal\_repos |
| Phenotype | cls.cls#OA\_versus\_normal\_repos |
| Upregulated in class | OA |
| GeneSet | KEGG\_PEROXISOME |
| Enrichment Score (ES) | 0.42443037 |
| Normalized Enrichment Score (NES) | 1.8740435 |
| Nominal p-value | 0.0 |
| FDR q-value | 0.0062228763 |
| FWER p-Value | 0.062 |
Table: GSEA Results Summary

  

Fig 1: Enrichment plot: KEGG\_PEROXISOME      
 Profile of the Running ES Score & Positions of GeneSet Members on the Rank Ordered List

  

| SYMBOL | TITLE | RANK IN GENE LIST | RANK METRIC SCORE | RUNNING ES | CORE ENRICHMENT || 1 | PEX3 | peroxisomal biogenesis factor 3 [Source:HGNC Symbol;Acc:HGNC:8858] | 11 | 1.178 | 0.0451 | Yes |
| 2 | PEX12 | peroxisomal biogenesis factor 12 [Source:HGNC Symbol;Acc:HGNC:8854] | 74 | 0.956 | 0.0776 | Yes |
| 3 | PEX2 | peroxisomal biogenesis factor 2 [Source:HGNC Symbol;Acc:HGNC:9717] | 94 | 0.921 | 0.1121 | Yes |
| 4 | SCP2 | sterol carrier protein 2 [Source:HGNC Symbol;Acc:HGNC:10606] | 105 | 0.905 | 0.1466 | Yes |
| 5 | MPV17 | mitochondrial inner membrane protein MPV17 [Source:HGNC Symbol;Acc:HGNC:7224] | 182 | 0.830 | 0.1731 | Yes |
| 6 | PEX11B | peroxisomal biogenesis factor 11 beta [Source:HGNC Symbol;Acc:HGNC:8853] | 266 | 0.777 | 0.1969 | Yes |
| 7 | PMVK | phosphomevalonate kinase [Source:HGNC Symbol;Acc:HGNC:9141] | 346 | 0.725 | 0.2191 | Yes |
| 8 | PEX7 | peroxisomal biogenesis factor 7 [Source:HGNC Symbol;Acc:HGNC:8860] | 467 | 0.673 | 0.2360 | Yes |
| 9 | GSTK1 | glutathione S-transferase kappa 1 [Source:HGNC Symbol;Acc:HGNC:16906] | 490 | 0.662 | 0.2601 | Yes |
| 10 | ECI2 | enoyl-CoA delta isomerase 2 [Source:HGNC Symbol;Acc:HGNC:14601] | 669 | 0.601 | 0.2697 | Yes |
| 11 | AMACR | alpha-methylacyl-CoA racemase [Source:HGNC Symbol;Acc:HGNC:451] | 749 | 0.581 | 0.2862 | Yes |
| 12 | IDH2 | isocitrate dehydrogenase (NADP(+)) 2 [Source:HGNC Symbol;Acc:HGNC:5383] | 967 | 0.525 | 0.2898 | Yes |
| 13 | GNPAT | glyceronephosphate O-acyltransferase [Source:HGNC Symbol;Acc:HGNC:4416] | 1026 | 0.512 | 0.3053 | Yes |
| 14 | ACOT8 | acyl-CoA thioesterase 8 [Source:HGNC Symbol;Acc:HGNC:15919] | 1088 | 0.500 | 0.3200 | Yes |
| 15 | PEX10 | peroxisomal biogenesis factor 10 [Source:HGNC Symbol;Acc:HGNC:8851] | 1253 | 0.467 | 0.3255 | Yes |
| 16 | HSD17B4 | hydroxysteroid 17-beta dehydrogenase 4 [Source:HGNC Symbol;Acc:HGNC:5213] | 1354 | 0.450 | 0.3352 | Yes |
| 17 | ACOX1 | acyl-CoA oxidase 1 [Source:HGNC Symbol;Acc:HGNC:119] | 1401 | 0.443 | 0.3489 | Yes |
| 18 | EPHX2 | epoxide hydrolase 2 [Source:HGNC Symbol;Acc:HGNC:3402] | 1659 | 0.405 | 0.3447 | Yes |
| 19 | HMGCL | 3-hydroxy-3-methylglutaryl-CoA lyase [Source:HGNC Symbol;Acc:HGNC:5005] | 1703 | 0.398 | 0.3569 | Yes |
| 20 | AGPS | alkylglycerone phosphate synthase [Source:HGNC Symbol;Acc:HGNC:327] | 1727 | 0.394 | 0.3705 | Yes |
| 21 | CAT | catalase [Source:HGNC Symbol;Acc:HGNC:1516] | 1731 | 0.394 | 0.3856 | Yes |
| 22 | DDO | D-aspartate oxidase [Source:HGNC Symbol;Acc:HGNC:2727] | 1839 | 0.380 | 0.3921 | Yes |
| 23 | ECH1 | enoyl-CoA hydratase 1 [Source:HGNC Symbol;Acc:HGNC:3149] | 1901 | 0.373 | 0.4019 | Yes |
| 24 | PECR | peroxisomal trans-2-enoyl-CoA reductase [Source:HGNC Symbol;Acc:HGNC:18281] | 1985 | 0.363 | 0.4096 | Yes |
| 25 | PEX19 | peroxisomal biogenesis factor 19 [Source:HGNC Symbol;Acc:HGNC:9713] | 2223 | 0.336 | 0.4042 | Yes |
| 26 | PHYH | phytanoyl-CoA 2-hydroxylase [Source:HGNC Symbol;Acc:HGNC:8940] | 2397 | 0.315 | 0.4030 | Yes |
| 27 | SOD1 | superoxide dismutase 1 [Source:HGNC Symbol;Acc:HGNC:11179] | 2413 | 0.313 | 0.4141 | Yes |
| 28 | CROT | carnitine O-octanoyltransferase [Source:HGNC Symbol;Acc:HGNC:2366] | 2567 | 0.295 | 0.4136 | Yes |
| 29 | DHRS4 | dehydrogenase/reductase 4 [Source:HGNC Symbol;Acc:HGNC:16985] | 2708 | 0.279 | 0.4136 | Yes |
| 30 | FAR2 | fatty acyl-CoA reductase 2 [Source:HGNC Symbol;Acc:HGNC:25531] | 2999 | 0.250 | 0.4008 | Yes |
| 31 | ABCD2 | ATP binding cassette subfamily D member 2 [Source:HGNC Symbol;Acc:HGNC:66] | 3031 | 0.246 | 0.4079 | Yes |
| 32 | PRDX1 | peroxiredoxin 1 [Source:HGNC Symbol;Acc:HGNC:9352] | 3113 | 0.239 | 0.4109 | Yes |
| 33 | CRAT | carnitine O-acetyltransferase [Source:HGNC Symbol;Acc:HGNC:2342] | 3159 | 0.234 | 0.4166 | Yes |
| 34 | ACOX2 | acyl-CoA oxidase 2 [Source:HGNC Symbol;Acc:HGNC:120] | 3176 | 0.233 | 0.4244 | Yes |
| 35 | HAO1 | hydroxyacid oxidase 1 [Source:HGNC Symbol;Acc:HGNC:4809] | 3493 | 0.203 | 0.4077 | No |
| 36 | PEX1 | peroxisomal biogenesis factor 1 [Source:HGNC Symbol;Acc:HGNC:8850] | 3655 | 0.190 | 0.4026 | No |
| 37 | PEX14 | peroxisomal biogenesis factor 14 [Source:HGNC Symbol;Acc:HGNC:8856] | 3985 | 0.157 | 0.3831 | No |
| 38 | PXMP2 | peroxisomal membrane protein 2 [Source:HGNC Symbol;Acc:HGNC:9716] | 4248 | 0.133 | 0.3678 | No |
| 39 | PXMP4 | peroxisomal membrane protein 4 [Source:HGNC Symbol;Acc:HGNC:15920] | 4455 | 0.117 | 0.3563 | No |
| 40 | MLYCD | malonyl-CoA decarboxylase [Source:HGNC Symbol;Acc:HGNC:7150] | 4852 | 0.083 | 0.3287 | No |
| 41 | SLC27A2 | solute carrier family 27 member 2 [Source:HGNC Symbol;Acc:HGNC:10996] | 5122 | 0.062 | 0.3102 | No |
| 42 | ACAA1 | acetyl-CoA acyltransferase 1 [Source:HGNC Symbol;Acc:HGNC:82] | 5175 | 0.058 | 0.3084 | No |
| 43 | PEX13 | peroxisomal biogenesis factor 13 [Source:HGNC Symbol;Acc:HGNC:8855] | 6228 | -0.023 | 0.2273 | No |
| 44 | DAO | D-amino acid oxidase [Source:HGNC Symbol;Acc:HGNC:2671] | 6252 | -0.024 | 0.2265 | No |
| 45 | PEX6 | peroxisomal biogenesis factor 6 [Source:HGNC Symbol;Acc:HGNC:8859] | 6325 | -0.030 | 0.2220 | No |
| 46 | ACSL6 | acyl-CoA synthetase long chain family member 6 [Source:HGNC Symbol;Acc:HGNC:16496] | 6505 | -0.043 | 0.2097 | No |
| 47 | ACOX3 | "acyl-CoA oxidase 3, pristanoyl [Source:HGNC Symbol;Acc:HGNC:121]" | 6747 | -0.059 | 0.1933 | No |
| 48 | PIPOX | pipecolic acid and sarcosine oxidase [Source:HGNC Symbol;Acc:HGNC:17804] | 6781 | -0.061 | 0.1931 | No |
| 49 | IDH1 | isocitrate dehydrogenase (NADP(+)) 1 [Source:HGNC Symbol;Acc:HGNC:5382] | 7109 | -0.089 | 0.1710 | No |
| 50 | PEX11A | peroxisomal biogenesis factor 11 alpha [Source:HGNC Symbol;Acc:HGNC:8852] | 7167 | -0.093 | 0.1702 | No |
| 51 | PAOX | polyamine oxidase [Source:HGNC Symbol;Acc:HGNC:20837] | 7212 | -0.097 | 0.1706 | No |
| 52 | DECR2 | "2,4-dienoyl-CoA reductase 2 [Source:HGNC Symbol;Acc:HGNC:2754]" | 7476 | -0.116 | 0.1546 | No |
| 53 | EHHADH | enoyl-CoA hydratase and 3-hydroxyacyl CoA dehydrogenase [Source:HGNC Symbol;Acc:HGNC:3247] | 7968 | -0.153 | 0.1223 | No |
| 54 | AGXT | alanine--glyoxylate and serine--pyruvate aminotransferase [Source:HGNC Symbol;Acc:HGNC:341] | 8115 | -0.164 | 0.1173 | No |
| 55 | ACSL3 | acyl-CoA synthetase long chain family member 3 [Source:HGNC Symbol;Acc:HGNC:3570] | 8170 | -0.169 | 0.1197 | No |
| 56 | XDH | xanthine dehydrogenase [Source:HGNC Symbol;Acc:HGNC:12805] | 8366 | -0.183 | 0.1116 | No |
| 57 | ABCD4 | ATP binding cassette subfamily D member 4 [Source:HGNC Symbol;Acc:HGNC:68] | 8652 | -0.205 | 0.0974 | No |
| 58 | ABCD3 | ATP binding cassette subfamily D member 3 [Source:HGNC Symbol;Acc:HGNC:67] | 8877 | -0.223 | 0.0887 | No |
| 59 | PEX16 | peroxisomal biogenesis factor 16 [Source:HGNC Symbol;Acc:HGNC:8857] | 8921 | -0.226 | 0.0941 | No |
| 60 | ABCD1 | ATP binding cassette subfamily D member 1 [Source:HGNC Symbol;Acc:HGNC:61] | 8930 | -0.226 | 0.1023 | No |
| 61 | ACSL5 | acyl-CoA synthetase long chain family member 5 [Source:HGNC Symbol;Acc:HGNC:16526] | 9136 | -0.243 | 0.0958 | No |
| 62 | PEX26 | peroxisomal biogenesis factor 26 [Source:HGNC Symbol;Acc:HGNC:22965] | 10303 | -0.351 | 0.0186 | No |
| 63 | BAAT | bile acid-CoA:amino acid N-acyltransferase [Source:HGNC Symbol;Acc:HGNC:932] | 10523 | -0.376 | 0.0162 | No |
| 64 | ACSL1 | acyl-CoA synthetase long chain family member 1 [Source:HGNC Symbol;Acc:HGNC:3569] | 10705 | -0.394 | 0.0175 | No |
| 65 | PEX5 | peroxisomal biogenesis factor 5 [Source:HGNC Symbol;Acc:HGNC:9719] | 10737 | -0.398 | 0.0306 | No |
| 66 | MVK | mevalonate kinase [Source:HGNC Symbol;Acc:HGNC:7530] | 10867 | -0.412 | 0.0367 | No |
| 67 | ACSL4 | acyl-CoA synthetase long chain family member 4 [Source:HGNC Symbol;Acc:HGNC:3571] | 11327 | -0.475 | 0.0194 | No |
| 68 | SLC25A17 | solute carrier family 25 member 17 [Source:HGNC Symbol;Acc:HGNC:10987] | 11669 | -0.527 | 0.0134 | No |
| 69 | HAO2 | hydroxyacid oxidase 2 [Source:HGNC Symbol;Acc:HGNC:4810] | 11987 | -0.591 | 0.0118 | No |
| 70 | NOS2 | nitric oxide synthase 2 [Source:HGNC Symbol;Acc:HGNC:7873] | 12262 | -0.665 | 0.0164 | No |
| 71 | SOD2 | superoxide dismutase 2 [Source:HGNC Symbol;Acc:HGNC:11180] | 12685 | -0.853 | 0.0168 | No |
Table: GSEA details [plain text format]

  

Fig 2: KEGG\_PEROXISOME      
 Blue-Pink O' Gram in the Space of the Analyzed GeneSet

  

Fig 3: KEGG\_PEROXISOME: Random ES distribution      
 Gene set null distribution of ES for **KEGG\_PEROXISOME**
